# Mitochondrial Cell‐Type‐Specific Profiling: Differential Function of Mesophyll and Guard Cells is Reflected by Their Mitochondrial Proteome

**DOI:** 10.1111/ppl.70579

**Published:** 2025-11-03

**Authors:** Friederike Hater, Jürgen Eirich, Paulina Heinkow, Saurabh Joshi, Yara Ehlert, Joakim Palovaara, Isil Erbasol Serbes, Amina Brahmia, Marco Diederichs, Sara Jalili, Nayanika Mukherjee, Paul Ssemanda, Annette Peter, Ole Schweser, Martin Kubitschke, Murali Krishna Madduri, Janine Kirstein, Kathrin Maedler, Olivia Andrea Masseck, Iris Finkemeier, Rita Groß‐Hardt

**Affiliations:** ^1^ Centre for Biomolecular Interactions University of Bremen Bremen Germany; ^2^ Institute of Plant Biology and Biotechnology University of Muenster Münster Germany; ^3^ Leibniz‐Institut für Alternsforschung Fritz‐Lipmann‐Institut e.V. (FLI) Jena Germany; ^4^ Institute for Biochemistry and Biophysics Friedrich Schiller Universität Jena Germany; ^5^ Institut für Zoologie University of Cologne Köln Germany

**Keywords:** cell‐type specific, mesophyll and guard cells, mitochondrial diversity, mRACE, proteome analysis

## Abstract

Cell specification and ensuing division of labor enable the accomplishment of complex tasks in multicellular organisms. In plants, mesophyll and guard cells exhibit striking functional, metabolic, and mechanophysical differences: mesophyll cells are highly specialized for efficient light‐energy conversion, whereas guard cells regulate gas exchange. Here, we address whether the cellular specialization is sustained by mitochondrial diversity between cells. We developed mRACE (RApid CEll‐type‐specific isolation of mitochondria), an innovative method for rapid cell‐type‐specific mitochondrial isolation. mRACE involves biotin labeling of mitochondria in target cells, followed by affinity‐based organelle enrichment with streptavidin‐coated microbeads or immobilization on functionalized glass slides. Using mitochondrial activity modulators, we confirmed that mRACE‐isolated mitochondria remain physiologically active, validating their suitability for downstream analyses. Applying mRACE, we isolated mitochondria from mesophyll and guard cells and performed a comprehensive proteome analysis. Notably, guard cells are enriched in active branched‐chain amino acid (BCAA) catabolism components, suggesting they exploit this unconventional carbon source for energy. Additionally, the two cell types differ in ribosomal protein composition and RNA‐editing proteins, highlighting mitochondrial specialization at multiple regulatory levels. We investigated gene activity for seven proteins differentially enriched between mesophyll and guard cells. Five promoter‐reporter constructs showed distinct activities between both cell types, validating our approach and indicating that distinct molecular make‐ups of guard and mesophyll mitochondria are substantially already established at the transcriptional level. Our findings reveal a new layer of mitochondrial specialization between mesophyll and guard cells, and establish mRACE as a powerful tool for isolating and analyzing mitochondria with cell‐type specificity.

## 1 Introduction

Multicellular organisms are powered by a plethora of dynamically interacting mitochondria. Beyond ATP production, mitochondria are involved in dozens of cellular processes, including signal transduction, redox homeostasis, and programmed cell death (Kuznetsov and Margreiter 2009; Martin et al. 2014; Scafaro et al. 2021; Gupta et al. 2022). Historically, mitochondria have largely been viewed as part of a rather homogeneous population. However, several studies have reported remarkable mitochondrial differences between tissues and even within individual cells (Kuznetsov and Margreiter 2009; Aryaman et al. 2019; Lu et al. 2019; Monzel et al. 2023). For example, in mice, liver mitochondria are enriched in pathways related to secondary metabolism and xenobiotic response, whereas brain mitochondria are primarily involved in protein targeting, the TCA cycle, and fission (MacDonald et al. 2019). Plant roots utilize different components of the tricarboxylic acid (TCA) cycle relative to shoots (Lee, Eubel, et al. 2011), and the plant egg cell lacks an aminoacyl‐tRNA synthetase present in the adjoining central cell (Kägi et al. 2010). The growing interest in understanding the prevalence and functional significance of mitochondrial diversity is reflected in the recent development of protocols for cell‐type‐specific mitochondrial isolation in plants (Boussardon et al. 2020; Lang et al. 2020; Niehaus et al. 2020) and in animals (Fecher et al. 2019; MacDonald et al. 2019). Some of these methods incorporate a pre‐sorting step, such as the physical separation of tissues and cells (e.g., Kang et al. (2024)). Other methods directly sort mitochondria based on fluorescence‐activated mitochondrial sorting (MacDonald et al. 2019), fluorophore‐targeted immunocapture (Fecher et al. 2019), or affinity purification (Boussardon et al. 2020).

Here, we have developed a rapid technique for selective purification of mitochondria (mRACE), based on the INTACT protocol for affinity‐based nuclei isolation (Deal and Henikoff 2010). mRACE enables mitochondrial purification using streptavidin‐coated magnetic microbeads or functionalized slides, the latter facilitating single‐mitochondrion analyses, potentially from specific cell types. For proof of concept, we chose to compare mesophyll and guard cell mitochondria as a test platform for three key reasons: (i) Mitochondria play a crucial role in photosynthetic cells and are essential for plant performance, (ii) they differ significantly in their molecular composition and mechanophysical properties, and (iii) since guard cells are two orders of magnitude less abundant than mesophyll cells, they are an ideal system for testing mRACE on cell populations of varying abundance.

Mesophyll cells are the epitome of plant source tissue, as they are specialized to maximize the light‐energy conversion during photosynthesis. In the light, they use ATP and NADPH to fix carbon dioxide (CO_2_) in chloroplasts into carbohydrates, which are then transported to sink plant tissues, such as roots, via the phloem. During the day, mesophyll cell mitochondria support chloroplast function by buffering metabolic processes, for example, by siphoning off excess NADPH and facilitating crucial steps of photorespiration. At night, they are the primary provider of ATP (Okada and Brennicke 2006; Igamberdiev 2020; Chadee et al. 2021). Guard cells are located in the epidermis of the leaf, where they operate as gatekeepers for the leaf's CO_2_ uptake by controlling the opening of the stomata via turgor pressure (Nilson and Assmann 2007; Daszkowska‐Golec and Szarejko 2013). To both maintain and rapidly adjust the turgor pressure via ATP‐dependent proton pumps, they demand high amounts of energy and are primarily consumers rather than producers of ATP (Giraud et al. 2009; Santelia and Lawson 2016; Flütsch, Wang, et al. 2020; Flütsch and Santelia 2021; Lim et al. 2022). In addition, guard cells exhibit a limited rate of photosynthesis. Instead, their mitochondria act as the main provider of ATP and guard cells were shown to be a direct recipient of mesophyll‐derived carbon sources (Lim et al. 2022; Flütsch, Nigro, et al. 2020). Therefore, despite their proximity, mesophyll and guard cells have different metabolic roles during the day, yet they work in concert to supply the plant with essential carbon compounds.

Using mRACE‐isolated mitochondria, we successfully performed tissue‐specific mitochondrial proteome analysis from root cultures, seedlings, and whole leaves, as well as cell type‐specific analysis from mesophyll and guard cells in *Arabidopsis thaliana* . Our results show intriguing discrepancies in the molecular make‐up of mesophyll and guard cell mitochondria, affecting different regulatory dimensions, including metabolism, posttranslational regulation, and RNA editing. Notably, in planta promoter‐reporter analysis of selected candidates aligns with these findings, underscoring the power of mRACE in identifying factors that are differentially employed by distinct cell types.

## 2 Material and Methods

### 2.1 Plants Growth Conditions

All plants are in *Arabidopsis thaliana* Col‐0 wildtype background. After stratification at 4°C for 2 days, plants were kept at 23°C in long‐day conditions (16 h light, 8 h darkness) to germinate. After shoot induction, plants were transferred to 18°C and long‐day conditions.

For root cultures, cultivation of seedlings and microscopic analysis, seeds were surface‐sterilized using ethanol (2× 70% EtOH, incubate 2–4 min, wash 2× 99.9% EtOH, air dry). Afterwards, seeds were sown on MS Medium [0.23% (w/v) MS basal salt, 0.05% (w/v) MES, 0.8% Phyto Agar, pH 5.9 with KOH] containing 25 μg/mL Kanamycin and 25 μg/mL phosphinothricin (PPT), if necessary for selection, and stratified at 4°C. For germination and until analysis/isolation, seedlings were kept at 23°C long‐day conditions.

Arabidopsis root cultures (ARC) were prepared as described by Czakó et al. (1993) with modifications. Approximately 40 7‐day‐old seedlings were transferred to ARC‐Medium [0.43% (w/v) MS Basal Salt, 0.3% (v/v) Miller's solution (6% (w/v) KH_2_PO_4_), 0.2% (v/v) Vitamix Stock, (0.5% (w/v) Thiaminhydrochloride, 0.05% (w/v) Pyridoxinhydrochloride, 0.1% (w/v) glycine, 0.05% (w/v) nicotic acid, 0.025% (w/v) folic acid, 0.05% (w/v) biotin), 0.02% (w/v) myo‐inositol, 3% (w/v) sucrose] in a sterile 500 mL flask. Cultures were incubated at 25°C and 80 rpm in darkness. After 3 weeks, the medium was exchanged for fresh ARC‐Medium. ARCs were used for mRACE after 5–6 weeks.

### 2.2 Cloning and Generation of Transgenic Plant Lines

#### 2.2.1 Generation of mRACE Lines

Both mRACE constructs were cloned based on the INTACT constructs (Deal and Henikoff 2010). The genomic sequence of the nuclear targeting fusion protein (NTF) was first subcloned into a pGIIBar vector (De Rybel et al. 2011). To generate the mitochondrial targeting fusion protein (MTF), the first 138 bp of *mtOM64* (AT5G09420), encoding the mitochondrial targeting domain (Lee, Lee, et al. 2011), were amplified from Arabidopsis gDNA with primers introducing a *Pac*I and a *Hin*dIII restriction site. The *eGFP* of the *NTF* was also amplified to introduce a *Hin*dIII restriction site at its 5′ end. The subcloned *pGIIBar_NTF* was restricted with *Pac*I and *Eco*RV, excising *WPP_eGFP*, and the newly amplified 138 bp of *mtOM64* and *eGFP* were introduced into the construct. The BirA was subcloned into a pGIIKan vector (De Rybel et al. 2011). For both constructs, a *p35S* promoter was introduced via *Asc*I/*Pac*I restriction sites.

Similarly, *pMYB60* and *pCAB3* were amplified according to Susek et al. (1993) and Cominelli et al. (2005) from Arabidopsis gDNA with additional *Asc*I and *Pac*I restriction sites. They were cloned into a pGEM‐T Vector (Promega) and then subcloned via the *Asc*I/*Pac*I restriction sites into a pGIIBar vector containing an *MTF* sequence. For the *pMYB60*‐ and *pCAB3*‐mRACE lines, a *pRPS5a* promoter was subcloned into a *pGIIKan_BirA* vector using *Asc*I and *Pvu*II restriction sites.

For localization analysis in HEK cells, the MTF insert was amplified by PCR, separated by gel electrophoresis, and cloned into an open N1 vector by isothermal In‐Fusion cloning in *E. coli* . Correct assembly was confirmed by restriction digestion (*Hin*dIII, *Not*I) and Sanger sequencing, which showed a 100% match with the designed construct. The resulting N1‐mAra vector was transfected into HEK cells.

Transformation of Arabidopsis was done using the *Agrobacteria‐*mediated transformation and the floral dip method (Clough and Bent 1998). Positive plant lines for *p35S::MTF* and *p35S::BirA* were crossed, as well as plants positive for *pRPS5a::BirA* and either *pMYB60::MTF* or *pCAB3::MTF*.

#### 2.2.2 Generation of Promoter‐Reporter Lines

For all constructs, the respective promoter region was amplified from Arabidopsis gDNA with primers introducing *Asc*I/*Pac*I restriction sites at the 3′‐and 5′‐ends and cloned into a pGEM‐T Vector (Promega). Using the newly introduced restriction sites, the promoters were then subcloned into an existing pGIIBar vector containing an *NLS_tdTomato_tNOS* sequence.

The fragments of the following lengths were amplified from upstream of the start codon: for *pGLDP2* 2064 bp, for *pAGT2* 860 bp, for *pAT4G17360* 717 bp, for *pAT5G47435* 1182 bp, for *pBCE2* 1333 bp, for *pERA2* 598 bp, and *pClpB‐m* 574 bp.

### 2.3 mRACE Purification of Mitochondria

#### 2.3.1 Bead‐Bound Isolation With mRACE

All steps were performed at 4°C or on ice. Material was homogenized with ice‐cold KPBS [136 mM KCl, 10 mM KH2PO4, pH 7.25 (Chen et *al*. 2017)] using either a mortar on ice or a homogenizer (Büchi) for seedling samples.

Homogenization was adapted to the starting material. For ARC samples, approx. Thirty grams of material was homogenized with 100 mL KPBS. The homogenate was filtered through four layers of sterile Gauze swabs (Hartmann AG). The remaining plant material was again added to the mortar, re‐homogenized with an additional 50 mL of ice‐cold KPBS, and filtered again through four layers of sterile Gauze swabs. For leaf samples, 4 g of material was harvested after 8 h of light. Leaves were placed in a mortar on ice and cut down into 5 mL ice‐cold KPBS using a scalpel. 0.5 g of Quartz sand and an additional 5 mL of ice‐cold KPBS were added, and the material was homogenized with the mortar. A prewetted (KPBS) 30 μm cell strainer (Sysmex) was used to filter the homogenate into a 50 mL tube. For the seedling samples, 400 mg of 7‐day‐old seedlings were homogenized with 300 μL ice‐cold KPBS using a homogenizer (Büchi). The homogenate was filtered through a 30 μm cell strainer (Sysmex). The homogenization tube was washed with 200 μL ice‐cold KPBS, and the remaining liquid was filtered into the homogenate as well.

Pre‐purification of the homogenates was achieved by two centrifugation steps: For the ARC samples, the homogenate was centrifuged at 2000 × g for 5 min at 4°C. The supernatant was carefully transferred to a new tube and centrifuged at 16,000× g for 10 min at 4°C. For leaf and seedling samples, the homogenate was then centrifuged at 2500× g for 5 min at 4°C. The supernatant was carefully transferred into a new tube and centrifuged at 15,000× g for 15 min at 4°C.

Mitochondria‐containing pellet was resuspended in 1 mL ice‐cold KPBS for ARC and leaf samples, and 100 μL ice‐cold KPBS for the seedling samples. Resuspended mitochondria were then added to 40 μL Dynabeads MyOne Streptavidin T1 (Thermo Fisher Scientific) in ice‐cold KPBS for ARC samples and to 50 or 20 μL of streptavidin‐coated Dynabeads T1 (Thermo Fisher Scientific) in ice‐cold KPBS for leaf and seedling samples, respectively.

Mitochondrial suspension was incubated with the beads at 10 rpm under overhead rotation for 10 min at 4°C. Afterwards, 1 mL ice‐cold KPBS was added to both ARC and leaf samples, whereas 800 μL ice‐cold KPBS was added to the seedling samples. Samples were then placed on a magnet for 2 min.

Washing steps were adjusted to the sample size. For ARC samples, pellets were successively washed on the magnet with 1.2 mL, 800, 500, 300, and 100 μL ice‐cold KPBS. For the leaf samples, pellets were washed in the magnet with 800, 2× 500, 300, 200, and 100 μL. For the seedling samples, pellets were washed on the magnet with 800, 500, 300, 200, and 100 μL. Finally, pellets were resuspended in 100 μL ice‐cold KPBS for all sample types.

#### 2.3.2 Affinity‐Base Immobilization With mRACE

**FIGURE 1:**
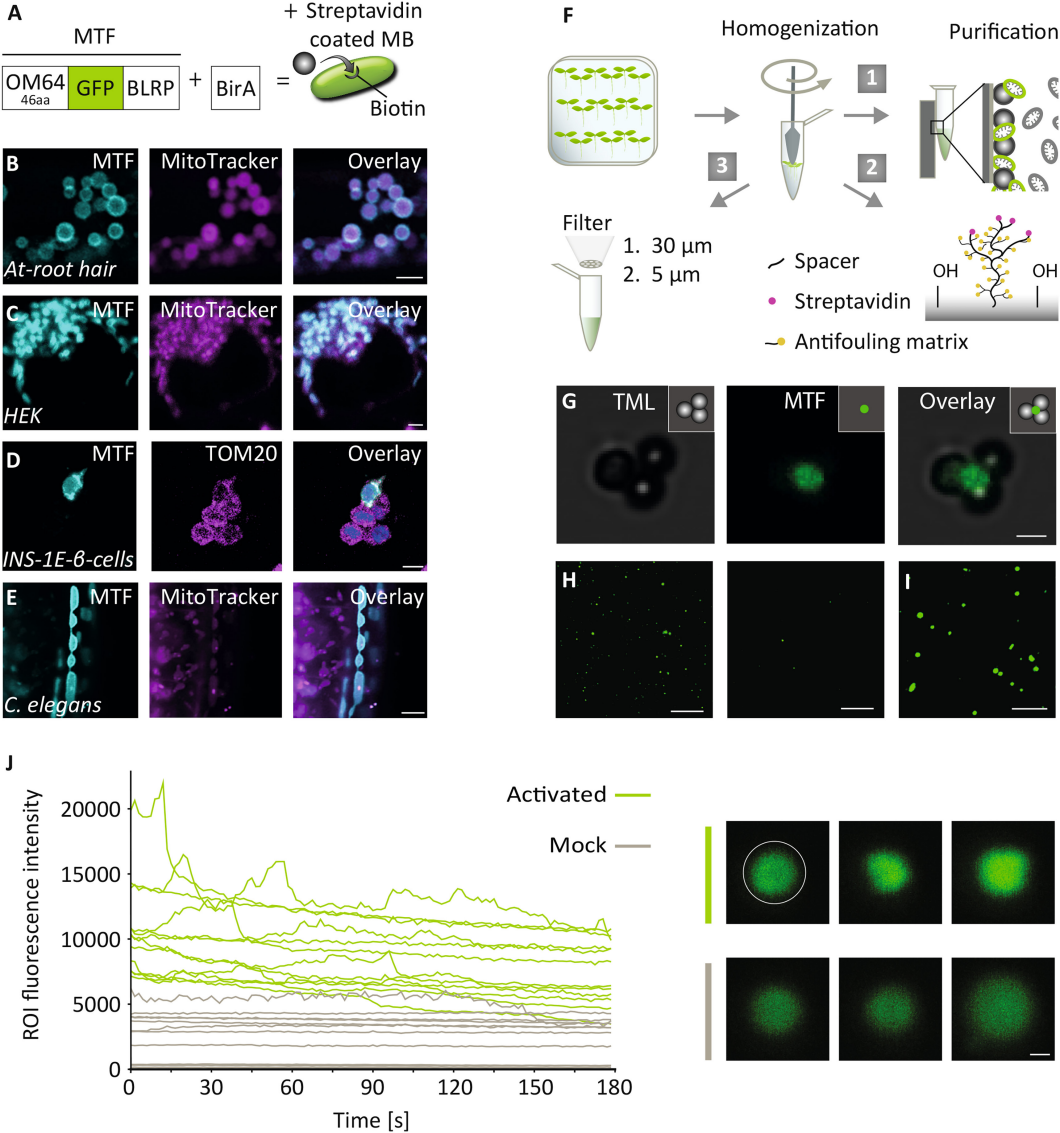
Exploiting mRACE for isolation and analysis of mitochondria from selective cell and tissue types. (A) The system capitalizes on the chimeric protein MTF, which contains the mitochondria‐targeting sequence of OM64, a GFP, and a biotin acceptor site. The latter gets biotinylated in the presence of the biotin ligase BirA. (B–E) mRACE‐based mitochondria tagging in organisms of different kingdoms. MTF was overexpressed and co‐expression of GFP and MitoTracker Red CMXRos or TOM20 is shown in single mitochondria of Arabidopsis (B), in the human embryonic kidney cell line (C), in the rat insulin‐producing β‐cell line INS‐1 with DAPI‐stained nuclei in the overlay (D; an example of five cells is shown, of which only one cell overexpressed MTF, whereas TOM20 remained stable in all cells), and in *C. elegans* (E). (F) F1 shows bead‐bound capture and isolation of biotinylated mitochondria using a magnet. F2 shows affinity‐based immobilization of biotinylated mitochondria using streptavidin‐coated chips. F3 shows rapid isolation of fluorescence‐tagged mitochondria using two sequential filtering steps (30, 5 μm) which can be followed by on‐chip‐immobilization via centrifugation. (G) Transmission light (left), fluorescence light (middle) and overlay channels (right) images showing bead‐bound mitochondria isolated from Arabidopsis roots expressing *p35S::MTF* and *p35S::BirA* using strategy F1. (H) Enrichment of biotinylated mitochondria immobilized on streptavidin‐coated chips using strategy F2: *p35S::MTF* and *p35S::BirA* expressing sample (left), *p35S::MTF* sample (control) (right). (I) Enrichment of sequentially filtered MTF‐tagged mitochondria (strategy F3) immobilized on a chip by centrifugation. (J) Representative activity measurements of individual cpYFP‐tagged mitochondria enriched with strategy F3 and immobilized on a chip by centrifugation. Quantification of the fluorescence intensities of individual cpYFP‐labeled mitochondria after activation with 10 mM succinate, 0.25 mM ATP and 20 μM rotenone (green) or mock treatment with basal incubation medium (beige) (left) and exemplary cpYFP‐signals of six individual mitochondria upon either activation or mock treatment (right). Scale bar 2 μm (B, C, E), 10 μm (D), 1 μm (G), 10 μm (H, I), 0.2 μm (J).

All steps were performed at 4°C or on ice. Homogenization, filtration, and centrifugation for mitochondrial enrichment from approx. Forty milligram of seedlings (~14 days old) were performed as described for bead‐bound isolation for seedlings. Resulting pellets were resuspended in 400 μL of KPBS buffer, and 100 μL of the sample was added to a pre‐cut (with diamond knife) PolyAn covalently coated streptavidin chip. Samples were incubated at 4°C for 10 min. Chips were washed 5× using 3 mL KPBS buffer in a 35 mm Petri plate on a shaker at 300 rpm for 1 min each. For imaging, chips were carefully placed on a μ‐Dish 35 mm, low (IBIDI GmbH) imaging dish containing KPBS buffer, and images were captured using a Leica DMI6000b epifluorescence inverted microscope equipped with a GFP filter cube. Images (Figure 1H) were equally processed in the built‐in Leica LAS AF software.

#### 2.3.3 mRACE Mitochondrial Filtration and Unspecific Immobilization

All steps were performed at 4°C or on ice. Roots from ~20 seedlings (~14 days old) were harvested and collected in a 1.5 mL Eppendorf tube containing 400 μL of 1× KPBS buffer. The root tissues were crushed open using a homogenizer (Büchi) to release organelles by mechanical lysis of cells for 2 min. The crude cell lysate was filtered through a pore size of 30 μm diameter cell strainer (Sysmex). The collected flow‐through was filtered through a fine pore size of 5 μm diameter cell strainer (pluriSelect) by successive centrifugation steps with a gradual increase in force (1 min at 60 × g, 30 s at 94 × g, 30 s at 211 × g, 1 min at 376 × g). Mitochondrial immobilization was adapted from Schwarzländer et al. (2011). In brief, the subsequent filtrates containing MTF‐labeled mitochondria were immobilized on a clean round 24 mm diameter coverslip by centrifugation (800 × g for 5 min at 4°C) in 1× KPBS buffer. Immobilized mitochondria (Figure 1I,J) were imaged using an epifluorescence microscope (Zeiss Axiovert 135 TV). Fluorescence images were processed in the built‐in JPK data processing software.

### 2.4 Quantification of MTF‐Positive Particles After Bead‐Bound mRACE Isolation

To quantify MTF‐positive particles in the purified sample, 5 μL were loaded onto a Neubauer counting chamber (Neubauer‐improved with dark lines, special depth 0.02 mm, Paul Marienfeld). Five of the 25 medium squares of the counting grid were captured (Leica DMI 6000 B microscope, DFC310FX camera, LAS AF software, Leica Microsystems), and particles expressing GFP were counted. The mean value (*n*) was taken and used to calculate the number of MTF‐positive particles in a 100 μL sample using the following formula:

$$\begin{array}{c} \mathrm{MTF}-\mathrm{positive}\;\mathrm{particles}\;\mathrm{in}\;100\;\mathrm{\mu L}=\left(\frac{25*n}{1\;{\mathrm{mm}}^{2}*0.02\;\mathrm{mm}}\right)*100 \end{array}$$

### 2.5 LC–MS/MS and Data Analysis

Proteins were eluted by boiling the beads in 250 μL SDT buffer (4% SDS, 100 mM Tris pH 7.6, 100 mM DTT) for 5 min. The supernatant was transferred to a fresh tube. Proteins were cleaned up following the SP3 protocol (Hughes et al. 2019). After a first binding step to the magnetic beads, proteins were alkylated with 14 mM CAA in 50 mM TEAB for 30 min at RT. Proteins were bound again to the magnetic beads. Washing and digestion were performed as described in the original protocol. Samples were analysed using an EASY‐nLC 1200 (Thermo Fisher Scientific) coupled to a Q Exactive HF (QEHF) or Exploris 480 (E480) mass spectrometer (Thermo Fisher Scientific), respectively. Peptides were separated and sprayed with either a 17 cm frit‐less silica emitter (New Objective, 0.75 μm inner diameter), packed in‐house with reversed‐phase ReproSil‐Pur C_18_ AQ 1.9 μm resin (Dr. Maisch) for the ARC and seedling samples, or a 25 cm fused silica emitter (75 μm inner diameter, CoAnn Technologies) packed in‐house with ReproSil‐Pur 120 C_18_ AQ 1.9 μm (Dr. Maisch) for all leaf samples. The columns were constantly kept at 50°C. Peptides were eluted in 115 min applying a segmented linear gradient of 0%–98% solvent B (solvent A 0% ACN, 0.1% FA; solvent B 80% ACN, 0.1% FA) at a flow rate of 300 nL/min. For all leaf samples, 0.5 μg of peptides per sample were analysed using a stepped gradient of 0%–45% solvent B (80% ACN, 0.1% FA) in 60 min at 250 μL/min, or 0%–55% solvent B in 100 min at 300 μL/min, followed by wash steps.

Peptide survey mass spectra were acquired in the Orbitrap analyzer, with a resolution of 12,000 on the E480 and 60,000 in MS^1^ for the QEHF. A resolution of 15,000 for MS^2^ spectra was used on both instruments. The scanned mass range was 300 to 1750 m/z. The normalized collision energy was set to 25. Precursors were selected with an isolation window of 1.3 m/z. MS/MS spectra were acquired with a target value of 10^5^ ions, a maximum injection time of 55 ms, and a fixed first mass of m/z 100. Peptides were excluded from fragmentation for MS^2^ by dynamic exclusion for 30 s, preventing repeated selection of precursors. Peptides with a charge of +1, > +6, or peptides with an unassigned charge state were excluded from fragmentation.

Processing of raw data was performed using the MaxQuant software version 2.1.3.0 (Tyanova et al. 2016) with default settings. MS/MS spectra were assigned to the Araport11 protein database. During the search, sequences of 248 common contaminant proteins as well as decoy sequences were automatically added. Trypsin specificity was required and a maximum of two missed cleavages was allowed. Carbamidomethylation of cysteine residues was set as fixed, oxidation of methionine, deamidation, and protein N‐terminal acetylation as variable modifications. A false discovery rate of 1% for peptide spectrum matches and proteins was applied. In addition to the default settings, match between runs, as well as LFQ (Label‐free quantification) and iBAQ (intensity‐based absolute quantification) were enabled.

MaxQuant output tables were further processed in R. Potential contaminants and reverse hits were removed. Protein locations were extracted from the SUBAcon4 database (Hooper et al. 2017). Plots were done with the ggplot2 package.

LFQ intensities were log_2_‐transformed. Missing values were imputed using the impute QRILC function of the imputeLCMD package (Lazar et al. 2022) for wildtype samples. Protein groups were considered for further analysis if they were quantified in more than two replicates of the respective cell‐type‐specific mRACE samples. Differential enrichment analysis was performed using limma (Ritchie et al. 2015) to compare LFQ intensities of WT and the cell type‐specific mRACE samples. For a functional enrichment analysis, proteins with the respective difference of log_2_FC between the respective cell types were analysed via the respective function provided by the stringDB (Szklarczyk et al. 2023). To calculate the relative rank, an average of the iBAQ values over the replicates per tissue was calculated and ranked from 1 to *n*. This rank was normalized to *n* = 100%.

All MaxQuant output and the following results can be found in Tables S1–S4.

### 2.6 Microscopic Analysis

#### 2.6.1 Colocalization Experiment Arabidopsis

Seven‐day‐old seedlings were incubated with 100 nM MitoTracker Red CMXRos (Thermo Fisher Scientific) in liquid MS Medium [0.23% (w/v) MS basal salt, 0.05% (w/v) MES, pH 5.9 with KOH] for 30 min at 4°C in darkness. Afterwards, seedlings were incubated in MS medium for only 30 min at room temperature in darkness. Microscope slides were prepared by placing the seedlings in a drop of 10% glycerol and covering them with a cover slide. Pictures were taken using the Airyscan super resolution mode of the LSM 880 Indimo, AxioObserver (Zeiss) with the Plan‐Apochromat 40×/1.4 Oil DIC M27 objective, the MBS 488/543 beam splitter and with an excitation wavelength of 488 nm, 1.0%, and 543 nm, 1.0%. The pixel dwell time was 8.19 μs.

#### 2.6.2 Colocalization Experiment in HEK Cells

Human embryonic kidney cells transiently transfected with *MTF* were costained with MitoTracker Red FM. Confocal pictures were taken at an Airyscan LSM880 (Zeiss) with a 40× oil objective.

#### 2.6.3 Colocalization Experiments in INS‐1E‐β‐Cells

The rat β‐cell line INS‐1E was transiently transfected with *MTF* for 48 h using jetPRIME transfection reagent (#114‐75; Polyplus transfection) according to the manufacturer's instructions; jetPRIME‐plasmid‐DNA complexes were added to complete RPMI‐1640 medium. Efficient transfection was evaluated based on Western blot (not shown) and fluorescent microscopy.

Cells were fixed and costained with MitoTracker Red CMXRos (#M7512 Thermo Fisher Scientific) or with anti‐TOM20 Rabbit mAb (Cell Signaling Technology #42406) and counterstained using Cy3 conjugated donkey anti‐rabbit antibody (Jackson Immunoresearch Laboratories #711‐165‐152). Nuclei were labeled using Vectashield antifade medium with DAPI (Vector Laboratories Inc. #H‐1200‐10). Confocal pictures were taken at an Airyscan LSM880 (Zeiss) with a 40× Oil objective.

#### 2.6.4 Colocalization Experiment in *C. elegans*

*C. elegans* expressing the *MTF* and *BirA::mCherry* under the control of the *myo‐3* promoter were incubated with 5 μM MitoTracker Red CMXRos (Thermo Fisher Scientific) in M9 (21.6 mM Na_2_HPO_4_, 22 mM KH_2_PO_4_, 8.5 mM NaCl, 18.7 mM NH_4_Cl) supplemented with 2.5% OP50 *E. coli* culture for 22 h at 20°C in the dark. Nematodes were anesthetized with 250 mM sodium azide and mounted onto a slide with a 3% agarose pad. The slides were covered with a coverslip and image acquisition was the same as described above for Arabidopsis.

#### 2.6.5 Confirmation of Cell‐Type Specific MTF Expression

Three‐day‐old Arabidopsis seedlings were placed in a drop of 10% glycerol and covered with a cover slide. Analysis was performed using the Zeiss CLSM 880 Confocal Laser Scanning Microscope with an excitation wavelength of 488 nm and a detection range of 493–551 nm. The laser was set at 2.0% and the Plan‐Apochromat 20×/0.87 M27 lens was used.

#### 2.6.6 Promoter‐Reporter Analysis

Five‐ to seven‐day‐old Arabidopsis seedlings were placed in a drop of 10% glycerol and covered with a cover slide. Analysis was performed using the Zeiss CLSM 880 Confocal Laser Scanning Microscope with an excitation wavelength of 543 nm and a detection range of 553–615 nm. The laser was set at 5%, and the Plan‐Apochromat 20×/0.87 M27 lens was used. A minimum of three seedlings were analyzed per promoter‐reporter line. For individual lines, the number of guard cell (GC) and mesophyll cell (MC) analyzed was as follows: *pGLDP2*, GC = 28, MC = 30; *pAGT2*, GC = 32, MC = 29; *pAT4G17360*, GC = 26, MC = 36; *pAT5G47435*, GC = 22, MC = 14; *pBCE2*, GC = 10, MC = 9; *pERA2*, GC = 14, MC = 7; and *pClpBm*, GC = 18, MC = 8.

For promoter‐reporter lines with fluorescence signals in both cell types, we additionally measured fluorescence intensity. Nuclei were labeled in 8‐bit converted images and analyzed using mean grey values in ImageJ.

## 3 Results and Discussion

### 3.1 The Chimeric MTF Protein Constitutes a Universal Tool for Mitochondria Labeling

There is growing evidence that mitochondria differ at a morphological and molecular level between cells and even within individual cells and that these differences are biologically and pathophysiologically relevant (Kuznetsov and Margreiter 2009; Kägi et al. 2010; Aryaman et al. 2019; Lu et al. 2019; MacDonald et al. 2019; Monzel et al. 2023). With new tools now enabling the isolation of mitochondria from distinct cell types, the field is entering an exciting phase—reflected in this issue by the parallel efforts of three teams converging on this emerging biological frontier (Boussardon et al. 2025, Ditz et al. 2025, and this work).

To study subgroups of mitochondria, we developed mRACE for mitochondrial isolation from selected cell types. The method uses a multimodular mitochondrial‐targeted fusion protein (MTF) that integrates three key functions (Figure 1A). First, it includes the mitochondria‐targeting domain of mtOM64, which has been shown to effectively target fusion proteins to the outer mitochondrial membrane (OMM) in Arabidopsis (Lister et al. 2007; Lee, Lee, et al. 2011; Nickel et al. 2018). Second, it incorporates a green fluorescent protein (GFP), enabling visualization of tagged mitochondria in target cells and facilitating live‐cell imaging of mitochondrial dynamics. Third, it features a biotin ligase recognition site (BLRP), providing a target site for biotinylation. The system is complemented by a second construct encoding the biotin ligase BirA, which attaches biotin to the BLRP acceptor site, allowing for the isolation, enrichment, and/or immobilization of engineered mitochondria through a biotin–streptavidin interaction using streptavidin‐coated microbeads or chips (Figure 1F). In addition, the GFP tag of the MTF can be used to identify mitochondria of the targeted cell type in a very fast, non‐specific isolation approach, based upon differential sieving and immobilizing mitochondria via centrifugation onto a chip within 10 min.

During mRACE establishment, live cell imaging of MTF positive plants revealed dynamically moving and interacting fluorescent organelles. To confirm the identity of the organelles, we performed co‐staining with MitoTracker Red CMXRos, a cell‐permeable fluorescent dye that selectively labels mitochondria. While MitoTracker accumulated within the mitochondria, the fluorescent signal associated with the chimeric MTF protein was detected on their outer surface, confirming the localization of MTF proteins to the outer mitochondrial membrane (Figure 1B). Given the substantial conservation of the anchor domains of outer mitochondrial membrane (OMM) proteins across eukaryotic organisms (Kanaji et al. 2000; Waizenegger et al. 2003; Walther and Rapaport 2009), we investigated whether MTF could similarly be used to label mitochondria in non‐plant model organisms, such as *C. elegans* , human embryonic kidney (HEK) cells, and rat β‐cells (INS‐1E). Co‐staining experiments with MitoTracker Red CMXRos or an anti‐TOM20 antibody showed colocalization with MTF in all tested model organisms (Figure 1C–E). These findings underscore the broad versatility and applicability of the system across diverse eukaryotic kingdoms, highlighting its potential as a universal tool for mitochondrial labeling.

To evaluate whether mRACE could enrich mitochondria for various downstream applications, we generated Arabidopsis seedlings expressing MTF and BirA under the broadly active *p35S* promoter (*p35S::MTF*, *p35S::BirA*; Figure 1F–H).

### 3.2 mRACE‐Based Chip Immobilization of Intact, Physiologically Responsive Mitochondria

Besides affinity purification using streptavidin‐coated beads, mRACE can be used to immobilize single mitochondria on slides. We adapted the protocol in two dimensions. First, for affinity‐based single‐mitochondrion on‐chip analysis, we used Arabidopsis seedlings. Following homogenization, filtration, and centrifugation steps, mitochondria were further purified from debris using streptavidin‐coated chips, facilitating the immobilization of biotinylated mitochondria (Figure 1F2). Microscopic inspection confirmed the specific binding of MTF‐functionalized mitochondria from BirA‐expressing seedlings, whereas no binding was observed in non‐BirA‐expressing seedlings (Figure 1H). Second, we established a rapid protocol that allows enrichment of mitochondria from seedlings in just 10 min without affinity purification. The approach is based on filtering the MTF‐labeled mitochondria sequentially using 30 μm and 5 μm diameter cell strainers from a crude cell lysate (Figure 1F3), followed by chip‐immobilization by centrifugation as established previously by Schwarzländer et al. (2011). Depending on the prevalence of the cell type under investigation, this mRACE protocol also allows cell type‐specific analysis of single mitochondria based on their fluorescence (Figure 1I). We used a mitochondrially targeted pH sensor (Schwarzländer et al. 2011) to test whether such immobilized single mitochondria were still intact and able to respire with single mitochondrion resolution. Notably, differential treatment of chip‐immobilized cpYFP‐expressing mitochondria resulted in differential fluorescence intensities (Figure 3A): mitochondria supplemented with succinate, ATP, and rotenone gave a strong and dynamic fluorescent signal as compared to mock‐treated mitochondria, indicating that the organelles were metabolically responsive. Interestingly, some mitochondria displayed short, transient pulses while others displayed stronger, longer pulses. Future experiments will help to elucidate the prevalence and functional significance of this differential responsiveness to factors modulating the activity of mitochondria.

### 3.3 mRACE Efficiently Enriches the Mitochondrial Signatures of Source Tissues

In a second approach, streptavidin‐coated microbeads were applied for affinity‐based organelle enrichment, following the idea of Deal and Henikoff (2010) and using a protocol similar to one previously published for mitochondria (Boussardon et al. 2020). This approach recovered bead‐bound MTF‐tagged mitochondria (Figure 1G).

**FIGURE 2:**
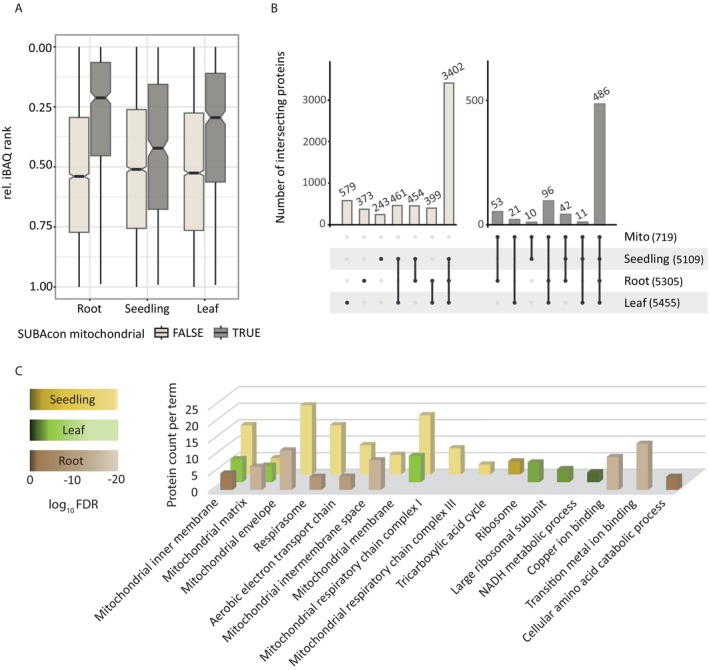
Proteomic analysis of mRACE‐isolated samples from Arabidopsis roots (ARC), leaves, and seedlings. (A) Plot showing the relative iBAQ rank of mitochondria‐associated proteins (grey) and non‐mitochondria‐associated proteins (beige). As a group, mitochondrial proteins rank higher, reflecting their enrichment in the samples. (B) Upset plot illustrating the intersection of non‐mitochondria (beige) and mitochondria‐associated proteins (grey) across the different samples. The number in brackets shows the number of proteins per term. (C) GO term enrichment analysis for the top 50 most abundant mitochondrial proteins in each sample set. Selected list of GO terms shown. Full list of GO terms is available in Table S2. Protein localization is based on SUBA4 predictions (Hooper et al. 2017). See sheet of Table S1 for iBAQ values.

To investigate whether and to what extent mRACE‐isolated mitochondria reveal mitochondrial diversity among different tissue types, we subjected mitochondria isolated from 30 g of Arabidopsis roots (Arabidopsis root culture; ARC), 4 g of leaves, and 400 mg of seedlings to an LC–MS/MS analysis. All tissue types expressed *p35S::MTF* and *p35S::BirA*. We identified 6630 protein groups throughout all runs, of which 719 protein groups were localized to mitochondria according to the SUBA4 database (Hooper et al. 2017) (Table S1). When comparing the relative iBAQ rank of mitochondrial versus non‐mitochondria‐associated proteins, mitochondrial proteins ranked higher; this implicates an enrichment of mitochondrial proteins in all three sample sets (Figure 2A).

Apart from mitochondrial‐associated proteins, proteins associated with the cytosol, plastid, and, to a lesser extent, the nucleus accumulate in all three samples (Table S1).

To determine whether the affinity purification is causative for the mitochondrial enrichment, we analyzed the intermediate isolation steps for the seedling samples. Compared to a mRACE negative control not carrying the mRACE constructs, both the pre‐bead sample and the supernatant revealed no enrichment of non‐mitochondria‐associated proteins (Figure S1). Thus, we concluded that the binding of biotinylated mitochondria to streptavidin‐coated microbeads was causative for the selective enrichment of mitochondrial proteins during affinity purification. Importantly, Gene Ontology (GO) assignments for the mitochondria‐associated proteins in the seedling samples detected proteins from all mitochondrial subcompartments, similar to Ditz et al. (2025) and Boussardon et al. (2025) (also part of this Special Issue), indicating that the isolation procedures recovered intact mitochondria (Figure S2).

Together, these findings highlight the robustness and versatility of mRACE as a powerful tool for the reliable enrichment of mitochondrial proteins.

When comparing the tissues, we found that 486 out of 719 mitochondrial proteins were shared across all three (Figure 2B). Ninety‐six protein groups were exclusively shared between leaf and root samples, 42 between seedling and root samples, and 11 between seedling and leaf samples. Fifty‐three protein groups were specific to the root samples, 21 for the leaf samples, and 10 for the seedling samples (Figure 2B). The high number of shared mitochondrial proteins between tissues is consistent with and further supports previous studies on tissue‐specific mitochondrial proteomes and transcriptomes, which have demonstrated a high similarity in mitochondrial composition between root and shoot (Lee et al. 2008; Lee, Eubel, et al. 2011).

We performed a functional enrichment analysis in StringDB (Szklarczyk et al. 2019), a database mapping protein–protein interactions, on the top 50 mitochondrial‐associated proteins for each tissue, selected based on an enrichment score of log_2_FC × −log_10_ (*p* value) (Figure 2C for highlighted GO terms, Table S2 for full data set). The sample set shows a similar enrichment of GO terms for mitochondrial compartments: “Mitochondrial inner membrane,” “Mitochondrial matrix,” “Mitochondrial envelope,” “Mitochondrial intermembrane space,” and “Mitochondrial membrane”. In addition, root and seedling samples share GO terms related to the mitochondrial electron transport chain. Within the seedling samples, proteins related to complex I, complex III, and TCA cycle are enriched, while proteins related to “Copper ion binding,” “Transition metal ion binding” and “cellular amino acid catabolic process” are enriched in the root samples. Surprisingly, the leaf samples show an enrichment of the GO terms “Ribosome” and “large ribosomal subunit” in addition to “NADH metabolic process,” suggesting heightened protein biosynthesis in leaf mitochondria. The absence of GO terms in the top 50 list related to aerobic respiration and TCA cycle activity in the leaf sample further supports the dominant role of photosynthesis in these tissues. Although classical markers of photorespiration, such as glycine decarboxylase (GDC), were notably absent from the functional enrichment analysis (with only two GDC‐related proteins among the top 50 mitochondrial proteins in both leaf and seedling samples), GDC‐related proteins were nonetheless enriched in leaf and, to a lesser extent, seedling samples compared to root samples (Table S1).

The functional enrichment analysis shows that mRACE is a powerful tool to detect the intricate differences distinguishing mitochondria from mature root tissues, mature leaves, and young seedlings.

### 3.4 Specific Purification of Mitochondria From Mesophyll and Guard Cells Using mRACE

As summarized above, mesophyll cells and guard cells exhibit striking functional, metabolic, and mechanical differences, with mesophyll cells being highly specialized for efficient light‐energy conversion, whereas guard cells orchestrate stomata opening, thereby controlling CO_2_ uptake and water management. To this end, it is not fully understood whether and how their distinct tasks are reflected by a functional specialization of their respective mitochondria, partly due to physical constraints hampering the isolation of these cell types.

**FIGURE 3:**
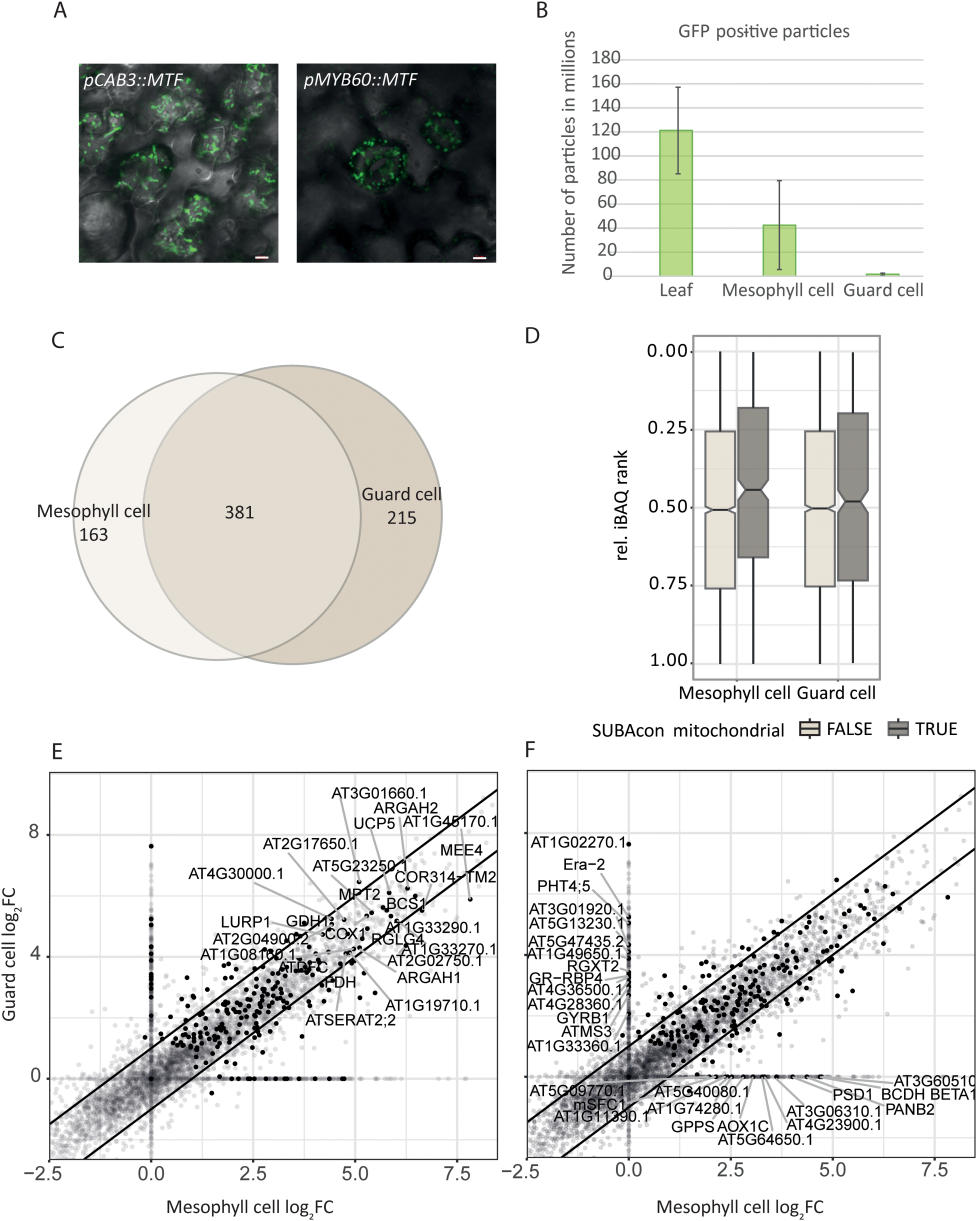
mRACE‐based isolation and LC–MS/MS analysis of mesophyll and guard cell mitochondria. (A) Cell‐type‐specific expression of *MTF* was achieved using *pCAB3* and *pMYB60* promoters; Scale bar 5 μm. (B) Quantification of GFP‐positive particles in whole leaf (*p35S*), mesophyll (*pCAB3*) and guard cell (*pMYB60*) samples. (C) Venn diagram of shared and unique mitochondrial protein groups in mesophyll (*pCAB3*) and guard cell (*pMYB60*) samples. (D) Mitochondria‐localized proteins [grey, SUBA4 (Hooper et al. 2017)] are enriched in both mesophyll (*pCAB3*) and guard cell (*pMYB60*) samples in comparison to the non‐mitochondrial proteome. (E and F) Scatterplot comparing log_2_FC of proteins (against a mRACE negative control) in mesophyll (*pCAB3*) and guard cell (*pMYB60*) samples. Mitochondria‐associated proteins [SUBA4; (Hooper et al. 2017)] are labeled in black. Both cell types share mitochondria‐associated proteins (E) but there are also noticeable differences between them (F). Experiments were conducted with four biological replicates for mesophyll (*pCAB3*) and five biological replicates for guard cell (*pMYB60*) samples. See Tables S1 and S3 for the respective log_2_FC values for the respective promoter lines.

To adopt the mRACE system for Arabidopsis mesophyll and guard cells, we made use of *CHLOROPHYLL A/B‐BINDING PROTEIN 3* and *MYB60* promoters (*pCAB3* and *pMYB60*, respectively), which have previously been shown to be specifically expressed in the respective cells (Susek et al. 1993; Cominelli et al. 2005). *pCAB3::MTF* and *pMYB60::MTF* plant lines were generated and crossed with a *pRPS5a::BirA* line. Plants positive for both constructs were subjected to live cell imaging. We detected fluorescence‐positive mitochondria in mesophyll and guard cells, respectively (Figure 3A), confirming cell‐type‐specific expression and the association of the chimeric MTF protein with the outer mitochondrial membrane.

Using mRACE, we purified bead‐bound mesophyll and guard cell mitochondria from Arabidopsis leaves. Quantification of mitochondria recovery yielded 1.65 million GFP‐positive particles for guard cells and 25 times more for mesophyll cells. In comparison, isolation of mitochondria from the whole leaf, using *p35S::MTF* and *p35S::BirA*, yielded 73 times more GFP‐positive particles than from the guard cell isolation (Figure 3B). Although we cannot rule out other interpretations like changes in promoter strength (Ditz et al. in this issue), this is likely to reflect the significantly different abundance of the respective cell types.

### 3.5 mRACE on Less Abundant Cell Types Enriches Mitochondria‐Associated Proteins

To investigate the mitochondrial proteome of mesophyll and guard cells, we subjected the respective mitochondrial isolates to LC–MS/MS analysis and compared them to a negative reference derived from seedlings lacking mRACE constructs. In total, 5181 protein groups were detected in mesophyll and guard cell samples (Table S1). Of these, 541 proteins are mitochondria‐localized [SUBA4; Hooper et al. 2017; Table S1]. In comparison to the abundance of all proteins, mitochondrial proteins show higher relative iBAQ ranks and are therefore enriched in all samples (Figure 3D). Notably, guard cell samples also exhibited enrichment of mitochondria‐associated proteins despite originating from a much less abundant cell type. This result underscores the remarkable efficiency of the mRACE approach in purifying mitochondria, even from rare cell types—a capability that highlights its potential for broader applications.

### 3.6 Mesophyll and Guard Cells Exhibit Distinct Mitochondrial Proteomes

When specifically comparing the mesophyll and guard cell samples to an mRACE negative seedling control, which did not contain any constructs, 544 mitochondria‐localized proteins (SUBA4: Hooper et al. 2017) are enriched in mesophyll samples, while 596 proteins are enriched in guard cell samples (Figure 3C, Table S3; Filters: log_2_FC > 3.3, *p* value (LIMMA) < 0.05). Among those, mesophyll and guard cell samples shared 381 proteins, while 163 and 215 proteins were solely enriched in one or the other (Figure 3C). Although both cell types share several mitochondria‐associated proteins (Figure 3C,E), there are noticeable differences between both cell types (Figure 3F). A more precise comparison, where we only considered proteins quantified in at least three replicates, revealed 50 proteins to be enriched in mesophyll (∆ log_2_FC > 1) and 48 proteins in guard cell (∆ log_2_FC < −1) samples.

To understand how these distinct mitochondrial proteomes enable mesophyll and guard cell activity, we subjected these 98 mitochondria‐associated protein groups to a functional enrichment analysis and a network analysis that predicts protein group associations (String DB; Szklarczyk et al. 2019) to assess putative cellular functions.

### 3.7 Mesophyll Cells Are a Dominant Contributor to the Whole Leaf Mitochondrial Proteome

**FIGURE 4:**
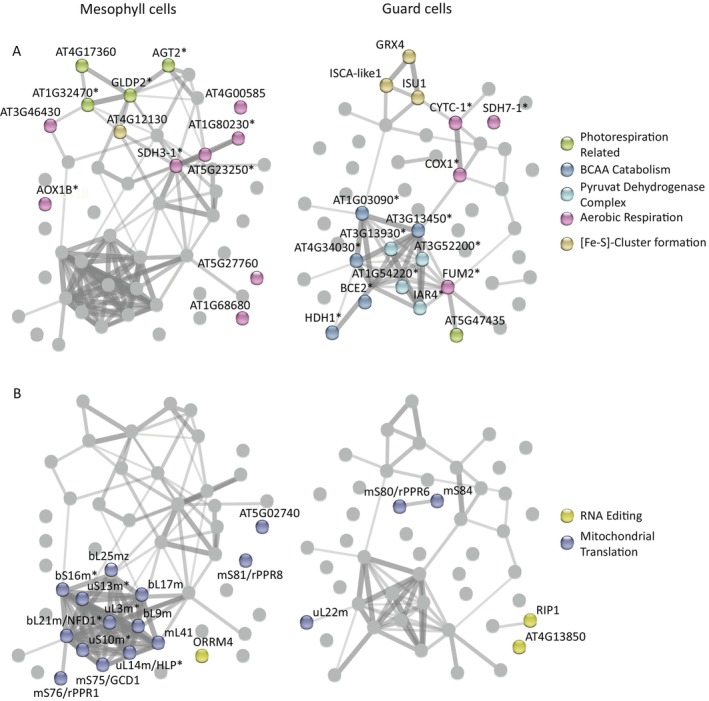
Network analysis of selected mitochondrial proteins in mesophyll and guard cells. Network analysis in StringDB of 50 proteins enriched in mesophyll cell (*pCAB3*) samples (∆ log_2_FC > 1) and 48 proteins enriched in guard cell (*pMYB60*) samples (∆ log_2_FC < −1) in at least three replicates. See Tables S1 and S3 for the respective log_2_FC values for the respective promoter lines. Each node represents a protein and the strength of edges indicates the strength of protein–protein association. (A) Protein groups related to cell‐type‐specific metabolic functions are highlighted. (B) Protein groups involved in mitochondrial protein biosynthesis are highlighted. Proteins indicated with an * were also identified in a functional enrichment analysis at StringDB: For proteins enriched in mesophyll cell samples these are “Glycine decarboxylation via glycine cleavage system” (*p* value 0.0147), “Glycine, serine and threonine metabolism” (*p* value 0.0145), “Aerobic respiration” (*p* value 0.0047), “Mitochondrial ribosome” (*p* value 8.16e‐06) and “Translation” (*p* value 0.0049). For proteins enriched in guard cell samples, these are “Pyruvate dehydrogenase complex” (*p* value 7.33e‐07), “Cellular response to sucrose starvation” (*p* value 0.0066), “Acetyl‐CoA biosynthetic processes from pyruvate” (*p* value 0.0170), “Branched‐chain amino acid catabolic process” (*p* value 0.00017) and “Aerobic respiration” (*p* value 0.0027).

Functional enrichment analysis of the 50 mitochondria‐associated proteins enriched in the mesophyll cell (*pCAB3*) samples showed that the most pronounced of the enriched GO terms was “Glycine decarboxylation via glycine cleavage system” (Figures 4 and S3), associated with the mitochondria‐localized part of photorespiration. Proteins involved in this process were previously shown to be differentially present in photosynthetic vs. non‐photosynthetic tissue and during the diurnal cycle within photosynthetic tissue (Lee et al. 2008, 2010; Lee, Eubel, et al. 2011).

Upon closer inspection, we identified additional protein groups involved in photorespiration and related processes, such as a 10‐formyl tetrahydrofolate deformylase (10‐FDF) (AT4G17360; Figure 4A), glycine decarboxylase complex P protein 1 (∆ log_2_FC of 0.82; Table S3), and alanine‐glyoxylate transaminase 2 (AGT2; Figure 4A). The latter is part of an alternative, mitochondrial‐localized biosynthesis pathway for glycine from glyoxylate (Niessen et al. 2007; Lee et al. 2008). AT4G12130, a member of the glycine decarboxylase complex T protein family, was also found to be enriched in mesophyll samples (Figure 4A), where it is likely involved in a folate‐dependent (Fe‐S) cluster metabolism (Waller et al. 2010, 2012). This aligns well with findings by Ditz et al. (2025) and Boussardon et al. (2025), who also observed a reduced abundance of photorespiration‐related proteins in guard cell mitochondria. Intriguingly, the second 10‐FDF present in mitochondria, AT5G47435, was not found in mesophyll cell samples but rather enriched in the guard cell (*pMYB60*) samples (∆ log_2_FC of −3.63; Figure 4A). The two 10‐FDF proteins function redundantly in photorespiration (Collakova et al. 2008). The overall reduced abundance of photorespiratory proteins in guard cell mitochondria (Figures 4A and S4; Table S3) aligns with their lower photosynthetic activity, indicating a tightly coordinated regulation between photorespiration and photosynthesis. However, this raises important questions about the dynamics of mitochondrial metabolism: To what extent do diurnal fluctuations observed in whole‐leaf mitochondrial proteomes (Lee et al. 2010) represent true metabolic shifts in mesophyll cells versus a mosaic of mitochondrial states across different cell types? These findings underscore the need for further investigations into how mitochondrial function is tailored to specific cell types and how this contributes to overall leaf metabolism.

The most pronounced GO term enriched among the 48 guard cell‐enriched mitochondrial proteins was “Cellular response to sucrose starvation” closely followed by “Leucine catabolism”, and “Branched‐chain amino acid metabolism” (Figures 4A and S4). Under sugar starvation, plant cells can utilize branched chain amino acid (BCAA) catabolism to feed electrons directly into the ubiquinone pool of the electron transport chain, producing substantial amounts of ATP—especially through leucine oxidation, which rivals glucose in energy yield (Ishizaki et al. 2005; Hildebrandt et al. 2015; Heinemann and Hildebrandt 2021). Although BCAA catabolism has been linked to starvation responses, particularly during the nighttime starvation phase in leaves (Lee et al. 2010), its role in guard cells under light conditions has not been explored. Our results suggest that guard cells, with their energy‐intensive functions, may utilize alternative energy sources, including amino acids, to meet their high ATP demands—a conclusion also independently reached by Ditz et al. (2025). Boussardon et al. (2025) reported a similar finding, but also noted a progressive accumulation of proteins associated with BCAA catabolism in mesophyll, vascular and guard cells in response to prolonged darkness, that is, a starvation episode for the plant (this issue). This aligns with the broader view of guard cells acting as a sink‐like tissue with a constant need for energy, as proposed by Flütsch, Nigro, et al. (2020) and Flütsch and Santelia (2021).

Supporting this, we find key components of the pyruvate dehydrogenase (PDH) family in the network analysis (Figure 4A), which indicate a highly active oxidative phosphorylation fueled by sugars and amino acid catabolism. In addition, the monothiol glutaredoxin GRXS15 and two further proteins involved in the mitochondrial biosynthesis of Fe‐S clusters are also enriched in guard cell samples (Figure 4A). GRXS15 was recently shown to be involved in pyruvate and BCAA degradation (Moseler et al. 2021), most likely via providing Fe‐S clusters for the production of lipoic cofactors, which both dehydrogenase complexes depend upon. Our data, therefore, suggest that guard cells consume not only sugars but also amino acids to fuel their high energy demand.

Our findings showcase that only the mesophyll mitochondria exhibit a proteome comparable to the published mitochondrial proteomes of whole leaf tissues, marking them as a dominant contributor to the whole leaf mitochondrial proteome. Guard cells, in comparison, display a mitochondrial proteome reminiscent of non‐photosynthetic or nocturnal leaf tissue, with an increase in PDH and BCAA catabolic protein abundances (Lee et al. 2008, 2010, 2012; Lee, Eubel, et al. 2011). Together, these findings point to a novel mechanism in guard cells: A dynamic adaptation to metabolic demands by consuming both sugars and amino acids to fuel their energy‐intensive functions. This dual reliance on carbohydrates and amino acids reflects the specialized metabolic strategies of guard cells to maintain their functionality under varying conditions, warranting further investigation into their unique bioenergetic pathways.

### 3.8 Differential Composition of the Mitochondrial Translation Machinery in Mesophyll and Guard Cells

Another prominent GO term enriched in mesophyll cells is “Mitochondrial ribosome” (Figures 4B and S3). Just as in the cytosol (Xue and Barna 2012; Petibon et al. 2021), the mitoribosome seems to work as a post‐transcriptional regulator (Kwasniak et al. 2013; Zhang et al. 2015; Robles and Quesada 2017; Grüttner et al. 2022). Compared to other eukaryotes, plant mitoribosomes harbor a more complex RNA and protein portfolio (Waltz et al. 2020). Among the ribosomal proteins, 16 are encoded by small gene families and are believed to have specialized functions as indicated by, for example, time and spatial specific expression (Skinner et al. 2001; Zhang et al. 2015; Robles and Quesada 2017). Mutant analysis of mitoribosome‐associated proteins showed a diverse set of phenotypes ranging from embryo lethal to no macroscopic difference, indicating that some components are essential for mitoribosome function, whereas other components are likely redundantly regulated or only integrated by a subset of mitoribosomes (Robles and Quesada 2017; Tomal et al. 2019; Waltz et al. 2020). In total, 14 protein groups enriched in mesophyll cell samples were associated with “Mitochondrial ribosomes”/“Mitochondrial Translation” (Figure S3 and Figure 4B), whereas three shared this association in the guard cell samples (Figure S4 and Figure 4B). Among the mesophyll‐enriched ribosomal proteins, seven are associated with the large (LSU) and six with the small ribosomal subunit (SSU) (Table S4, Figure 4). Of the seven LSU proteins, four have close paralogues with which they form small gene families (Robles and Quesada 2017; Tomal et al. 2019) (Table S4). One of them, *ul14m/HUELLENLOS PARALOGUE*, was found to be differentially expressed throughout the plant, with lower expression levels in the carpel, for example, where it is replaced by *HUELLENLOS* (Skinner et al. 2001). Similarly, a tissue‐specific expression pattern is also known for the mesophyll‐enriched SSU protein mS75 (also known as GAMETE CELL DEFECTIVE1, At5g62270), with the highest expression in the reproductive tissue, where it is crucial for the correct patterning of the gametophytes. Whether their differential presence in mesophyll and guard cell samples indicates a specialized function here remains an open question. The three ribosomal proteins enriched in guard cells comprise one LSU and two SSU proteins. The LSU protein is encoded by a gene of the small gene family *uL22m* (Table S4, Figure 4B). Taken together, our findings underscore a cell‐type‐specific composition of the translation machinery, suggesting that the translation machinery in guard and mesophyll cells differs in function, possibly to meet the unique demands of these distinct cell types, a hypothesis also proposed in Boussardon et al. (2025); this special issue.

Besides the cell‐type‐specific composition of the translation machinery, it is important to note that overall mitoribosomal proteins are enriched in the mesophyll sample (Tables S3 and S4, Figure 4B). Our data on mesophyll and guard cell mitochondrial proteomes suggest that only mesophyll cells are undergoing the diurnal shift described for photosynthetic tissues (Lee et al. 2010). It is therefore likely that they also need a much more regulated protein biosynthesis. However, it cannot be ruled out that this difference in the presence of mitoribosomal proteins is an artifact of the difference in mitochondria density in the two sample sets (Figure 3B). Nevertheless, protein groups, especially those with known paralogues or differential expression patterns, are potential targets for the cell type‐specific regulation of the mitochondrial proteome via the mitoribosome.

In addition to factors involved in translation, we identified three RNA editing factors specifically enriched in the mesophyll or guard cell samples. RNA editing is a post‐transcriptional modification process that involves the conversion of specific cytidines (C) to uridines (U) in RNA molecules. This modification helps prevent mutations in the coding sequence from being translated into defective proteins (Benne et al. 1986). The components of the RNA editosome can form both homo‐ and heterodimers, and each is typically specific to particular target sites in the RNA (Bentolila et al. 2013; Shi, Bentolila, and Hanson 2016; Shi, Germain, et al. 2016; Shi et al. 2017).

In the guard cell samples, RIP1 (MORF8) and ORRM5 (GR‐RBP2) are enriched (Figure 4B). RIP1 is dual‐targeted to both mitochondria and chloroplasts (Bentolila et al. 2012). *rip1* mutants display a severe dwarf phenotype, along with a significant reduction in C‐to‐U editing—20% in plastids and 75% in mitochondria—highlighting its crucial role in RNA editing across organelles (Bentolila et al. 2012, 2013). *ORRM5*, on the other hand, is involved in diverse developmental processes (Vermel et al. 2002; Fusaro et al. 2007; Czolpinska and Rurek 2018) and is the only known RNA editing factor reported to have a negative effect on RNA editing (Shi et al. 2017). By comparison, in the mesophyll cell samples, ORRM4 is enriched (Figure 4B). ORRM4 is necessary for editing 44% of the C‐to‐U editing sites in mitochondria, and its mutant showed stunted growth and delayed flowering (Shi, Germain, et al. 2016). Recently, an in‐depth study of the mitochondrial proteome of *Arabidopsis* cell cultures provided the first insight into the frequency of non‐ or only partially RNA‐edited mitochondrial proteins, noting specifically the high frequency among mitoribosomal proteins (Rugen et al. 2024). Our findings raise the intriguing possibility that RNA editing in mitochondria may be regulated in a cell‐type‐specific manner and that differential editing landscapes contribute to the functional specialization of the mitochondrial proteome in these cell types. This hypothesis is also supported by the findings of Boussardon et al. (2020), even though the abundance of RNA editing factors (especially MORF8, GRP23, NUWA, and DYW2) strongly decreased in mitochondria of guard cells in response to prolonged darkness.

### 3.9 Gene Expression Analysis Uncovers the Potential of mRACE for Identifying Cell‐Type‐Specific Mitochondrial Signatures

It was previously reported that there is a weak connection between transcriptome and proteome data recovered from tissue‐specific mitochondria analysis (Lee, Eubel, et al. 2011). To assess the predictive power of the mRACE approach in identifying genes encoding cell‐type‐specific mitochondrial proteins, we characterized the promoter activity of several metabolic proteins identified in the mesophyll versus guard cell comparison. We generated promoter‐tdTomato fusion constructs for proteins related to photorespiration (*pGLDP2*, *pAGT2*, *pAT4G17360*, and *pAT5G47435*) and BCAA catabolism (*pbce2*). GLDP2, AGT2, and AT4G17360 were shown by proteomic analysis to be enriched in mesophyll cell samples, whereas AT5G47435 and bce2 were enriched in guard cell samples (Figure 4 and Table S3). Considering that the genomic context of a T‐DNA insertion may influence apparent promoter activity, we verified and confirmed that the observed expression patterns were reproducible across two independent transgenic lines.

**FIGURE 5:**
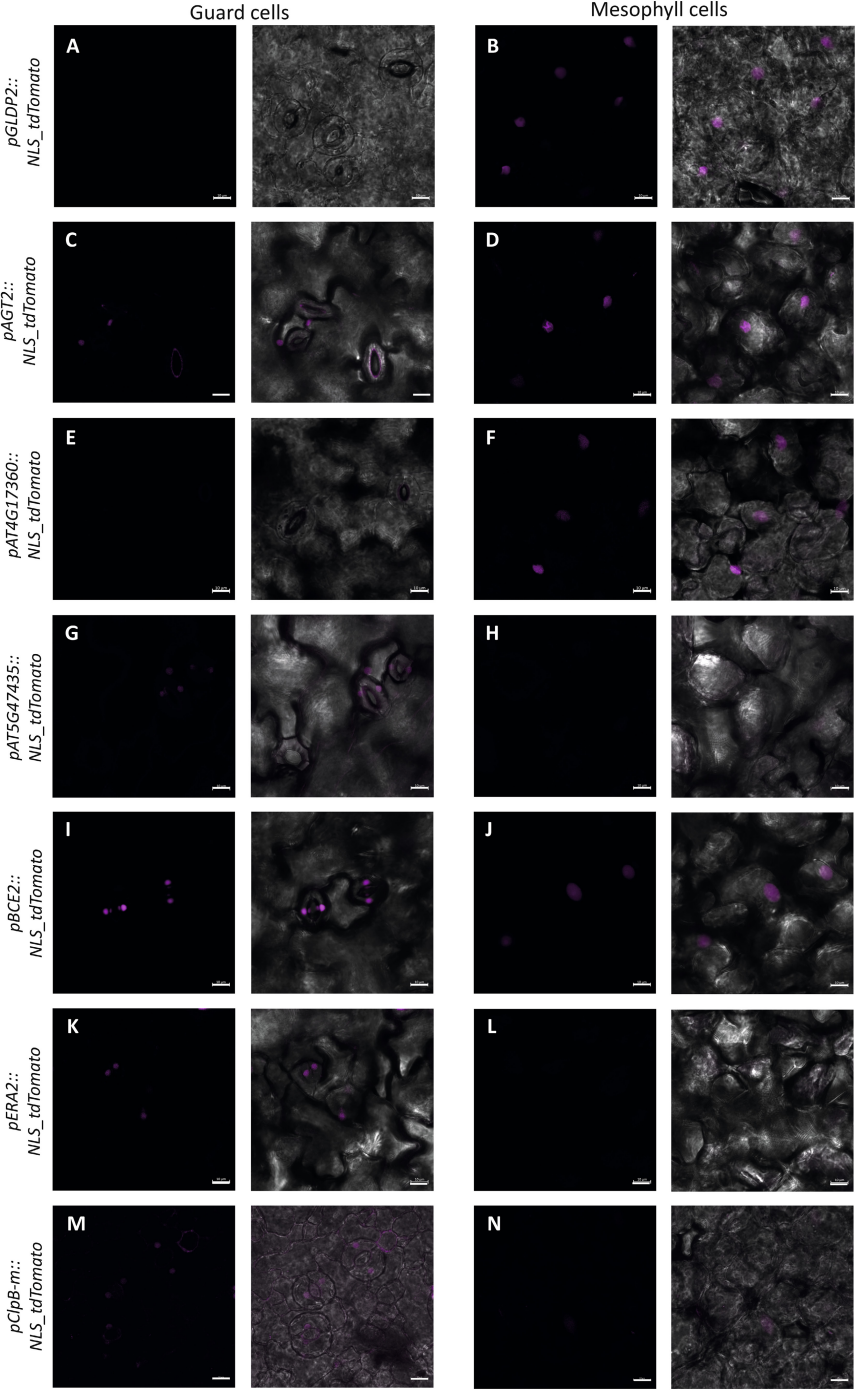
Promoter‐reporter analysis of candidate genes. Cotyledons in 5–7‐day‐old seedlings of plants expressing an *NLS_tdTomato* reporter under the respective promoter. A minimum of three seedlings were analyzed per promoter‐reporter line. For individual lines, the number of guard cell (GC) and mesophyll cell (MC) analyzed were as follows: *pGLDP2* (A,B), GC = 28, MC = 30; *pAGT2* (C,D), GC = 32, MC = 29; *pAT4G17360* (E,F), GC = 26, MC = 36; *pAT5G47435* (G,H), GC = 22, MC = 14; *pBCE2* (I,J), GC = 10, MC = 9; *pERA2* (K,L), GC = 14, MC = 7; and *pClpB‐m* (M,N), GC = 18, MC = 8. Scale bar is 10 μm.

The promoter analysis revealed distinct patterns of activity. Notably, *pGLDP2, pAGT2*, and *pAT4G17360* showed a clear and consistent promoter signal in mesophyll cells, with no detectable activity in guard cells (Figure 5A–F). These results are in line with the proteomic data, which suggest that these proteins are involved in mesophyll‐specific mitochondrial functions. Conversely, *pAT5G47435* exhibited active promoter expression in guard cells but not in mesophyll cells (Figure 5G,H). This finding strongly supports the hypothesis that the two 10‐FDF proteins (AT4G17360 and AT5G47435) have a specialized subcellular role, potentially highlighting their divergent functions in different cell types.

Both *pAGT2* and *pAT5G47435* also showed expression in pavement cells adjacent to guard cells (Figure 5C,G). Notably, *pAGT2* displayed infrequent activity in guard cells up to day 4 and lost activity in mesophyll cells after Day 7 (data not shown). These observations point to a complex temporal and spatial regulation of gene expression.

In contrast, *pBCE2* showed a less distinct pattern. Promoter activity could be detected in both guard and mesophyll cells (Figure 5I,J). However, the signal was significantly stronger in guard cells (Figures 5I,J and S5A). BCE2 is part of the BCAA catabolism and, according to previous data on the diurnal shift in photosynthetic tissues, expression peaks during early morning (Lee et al. 2010). Since the promoter analysis was performed after approximately 8 h of light, this differential signal intensity might reflect a prolonged promoter activity during daylight in guard cells, indicating that these cells could rely on certain mitochondrial processes for an extended period throughout the day.

In addition, we analyzed promoter activity of the mitoribosomal ripening factor ERA2 (Suwastika et al. 2014; Cheng et al. 2018) and the mitochondrial chaperone ClpB‐m (Lee et al. 2007; Singh and Grover 2010). Both were shown by proteomics data to be enriched in the guard cell samples (Table S3). Although ERA2 was found exclusively in guard cells (∆ log_2_FC −5.40), ClpB‐m was found in both cell types, but was detected at a much higher rate in guard cells with a ∆ log_2_FC of −2.01. We observed *pERA2* activity in guard cells starting from 5‐day‐old seedlings, but no activity in mesophyll cells (Figure 5K,L). Interestingly, approximately half of the guard cells in the cotyledons showed no *pERA2* activity, suggesting differentiated regulation among individual guard cells. For *pClpB‐m*, we observed strong expression in guard cells and significantly weaker expression in mesophyll cells (Figures 5M,N and S5B), as predicted by the cell‐type‐specific proteomic data.

Our finding that five out of seven promoter‐reporter constructs reflect the differential protein enrichment detected by LC–MS/LC‐MMS indicates that the molecular asymmetries between mesophyll and guard cell mitochondria are, to a substantial extent, already established at the transcriptional level. In addition, they underscore the power of the mRACE approach in identifying cell‐type‐specific protein signatures and its predictive force for uncovering mitochondria‐associated gene functions that are regulated in a cell‐type‐specific manner.

mRACE, along with other isolation protocols targeting mitochondrial subpopulations, contributes to unlocking new dimensions of mitochondrial diversity that conventional methods often overlook. Our results reveal a new layer of mitochondrial specialization between mesophyll and guard cells and establish mRACE as a powerful tool for isolating and analyzing mitochondria with cell‐type specificity. This more refined view is particularly relevant, as plant mitochondria influence agronomically important traits such as fertility, plant vigor, chloroplast function, and cross‐compatibility (Chevigny et al. 2020).

## Author Contributions

Friederike Hater, Rita Groß‐Hardt, Jürgen Eirich, and Iris Finkemeier conceived the experiments, analyzed the data, and wrote the manuscript. Jürgen Eirich, Paulina Heinkow, and Iris Finkemeier performed the LC–MS/MS experiments and pre‐assessment of the proteome data. Saurabh Joshi and Yara Ehlert conducted the mRACE filtration and mitochondria‐on‐chip experiments, with Joakim Palovaara conceiving the mRACE filtration system. Friederike Hater, Joakim Palovaara, Yara Ehlert, Isil Erbasol Serbes, Amina Brahmia, Marco Diederichs, Sara Jalili, Nayanika Mukherjee, Paul Ssemanda, Ole Schweser, Martin Kubitschke, and Murali Krishna Madduri contributed to the generation of constructs and isolation of mitochondria. Janine Kirstein, Kathrin Maedler, Annette Peter, and Olivia Andrea Masseck, along with their respective teams, demonstrated that MTF is targeted to the outer mitochondrial membrane in other model organisms. Saurabh Joshi contributed to writing parts of the manuscript.

## Supporting information


**Data S1:** ppl70579‐sup‐0001‐Supinfo.pdf.

**Table S1:** ppl70579‐sup‐0001‐TableS1.xlsx.

**Table S2:** ppl70579‐sup‐0002‐TableS2.xlsx.

**Table S3:** ppl70579‐sup‐0003‐TableS3.xlsx.

## Data Availability

Mass spectrometry raw data and search result files are available via the jPOST repository (jPOSTrepo) and can be accessed under the following links: https://repository.jpostdb.org/entry/JPST001156. https://repository.jpostdb.org/entry/JPST003686.
