# Supplementary material for: Mitochondrial Cell‐Type‐Specific Profiling: Differential Function of Mesophyll and Guard Cells is Reflected by Their Mitochondrial Proteome
Source: Physiol Plant. 2025 Nov 3;177(6):e70579. doi: 10.1111/ppl.70579 (PMC12581003; doi:10.1111/ppl.70579)
Supplement: Supplementary file 1 — Data S1: ppl70579‐sup‐0001‐Supinfo.pdf. [file PPL-177-e70579-s004.pdf]

Supplements

Mitochondrial cell-type-specific profiling: Differential function of mesophyll and guard cells is reflected by their mitochondrial proteome

Friederike Hater*[1], Jürgen Eirich[2], Paulina Heinkow[2], Saurabh Joshi[1], Yara Ehlert[1], Joakim Palovaara[1], Isil Erbasol Serbes[1], Amina Brahmia[1], Marco Diederichs[1], Sara Jalili[1], Nayanika Mukherjee[1], Paul Ssemanda[1], Annette Peter[1], Ole Schweser[1], Martin Kubitschke[1], Murali Krishna Madduri[1], Janine Kirstein[1,6,7], Kathrin Maedler[1], Olivia Andrea Masseck[1,8], Iris Finkemeier[2] and Rita Groß-Hardt*[1]

[1]Centre for Biomolecular Interactions, University of Bremen, Leobener Strasse 5, 28395 Bremen, Germany

[2]Institute of Plant Biology and Biotechnology, University of Muenster, Schlossplatz 7, 48149 Münster, Germany

[6]Leibniz-Institut für Alternsforschung, Fritz-Lipmann-Institut e.V. (FLI), Beutenbergstraße 11, 07745 Jena, Germany

[7]Institute for Biochemistry and Biophysics, Friedrich Schiller Universität, Hans Knöll Strasse 2, 07745 Jena, Germany

[8]Institut für Zoologie, University of Cologne, Zülpicher Strasse 47b, 50674 Köln, Germany

*Corresponding authors:

Rita Groß-Hardt (gross-hardt@uni-bremen.de)

Friederike Hater (friederike.hater@uni-bremen.de)

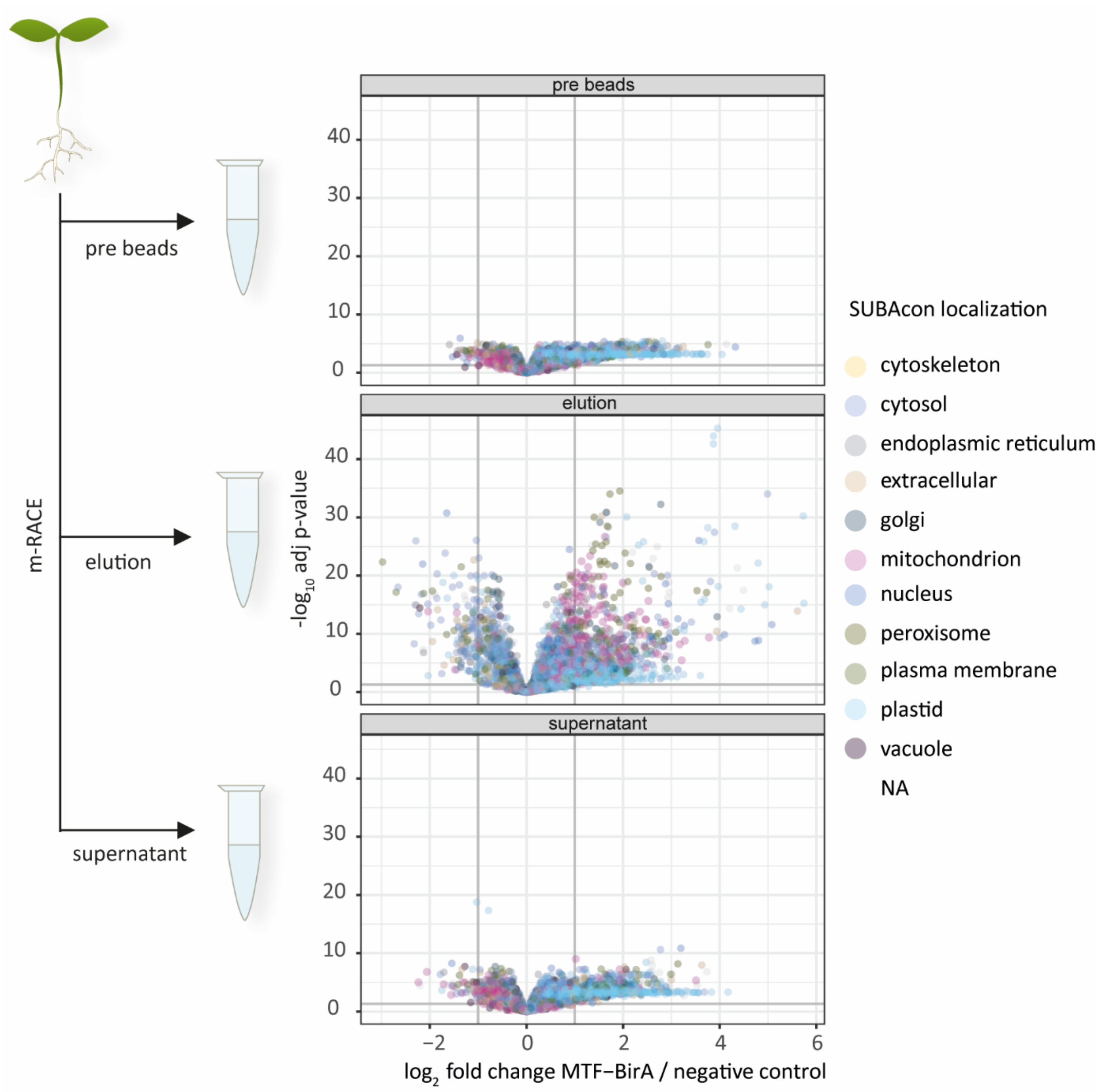

Figure S1: Enrichment of mitochondria-associated proteins during mRACE bead-based isolation of mitochondria depicted in three stages: pre-bead, bead-bound (eluate), and supernatant**.** Volcano plots show the relationship between the log_2 fold-change of samples relative to a control and the -log_{10} adjusted P-Value (LIMMA analysis). Aliquots of 100 µL for the three stages were used for LC-MS/MS analysis. Negative control samples, which lack expression of MTF or BirA, were collected at the same three stages. Neither pre-bead nor supernatant samples show enrichment of mitochondrial proteins compared to the control. However, there is a significant enrichment of mitochondrial proteins in the eluate.

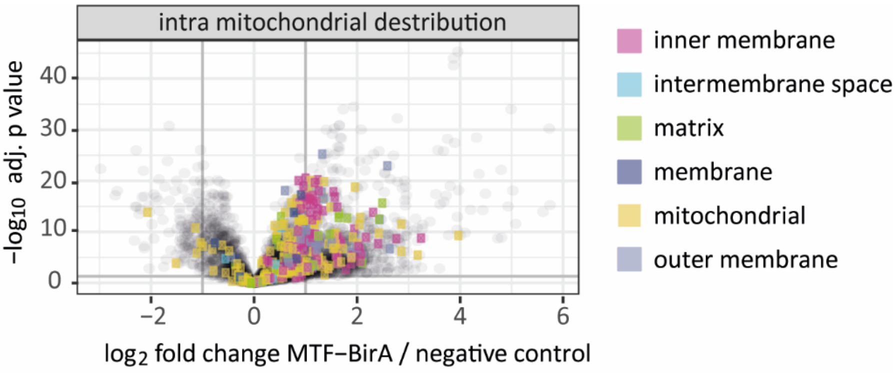

Figure S2: Intra-mitochondrial distribution of proteins. Volcano plot comparing SUBAcon-designated localization of mitochondria-associated proteins in seedling samples to a negative control, that did not express MTF or BirA.

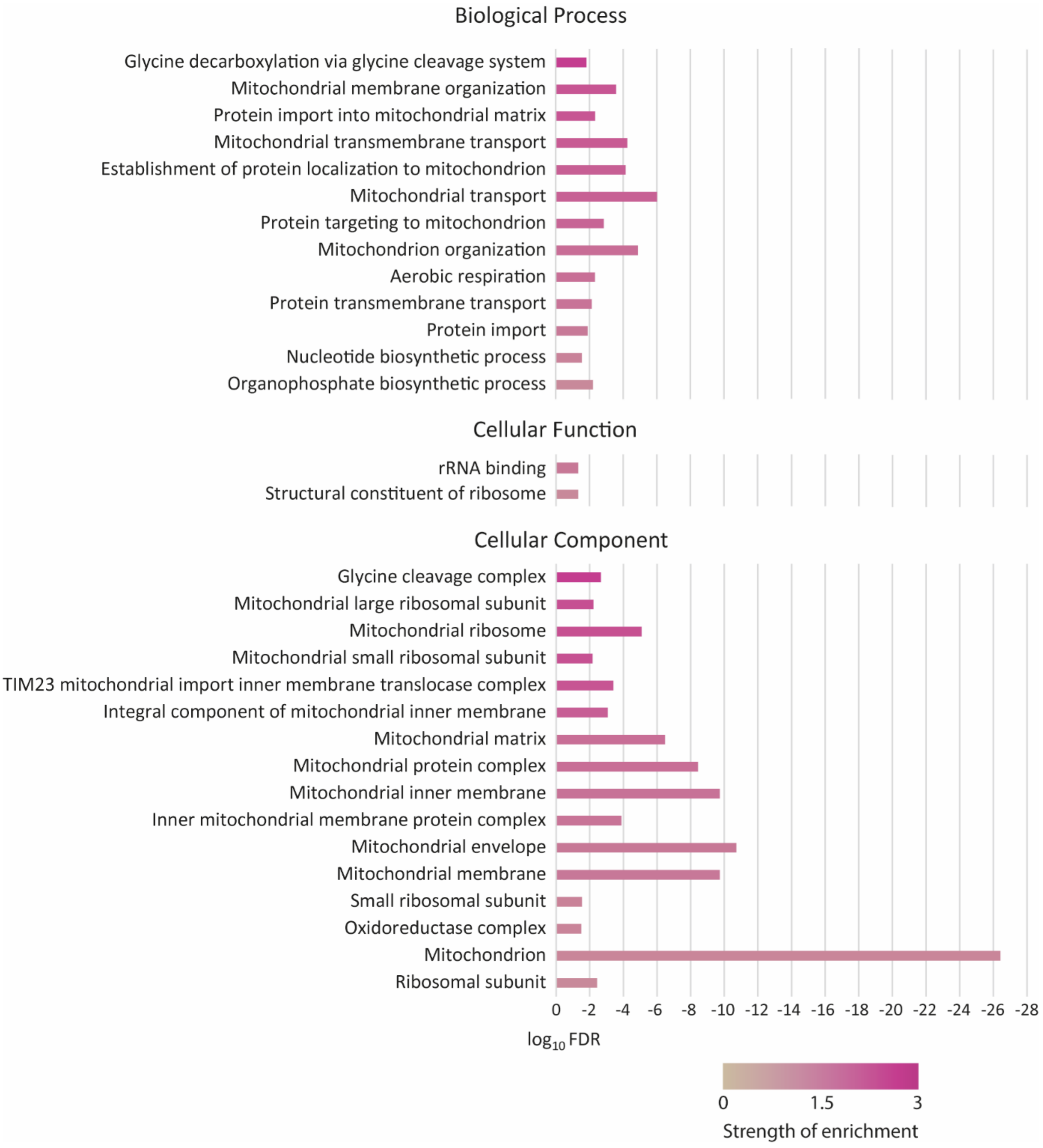

Figure S3: Functional enrichment analysis of mesophyll-enriched proteins. 50 mesophyll-enriched proteins (Δlog_2 FC >1, identified in at least three replicates) were subjected to GO term enrichment analysis on String DB. Only GO terms with a strength of enrichment (log_{10}(observed/expected number of proteins in a random set)) >1 are shown.

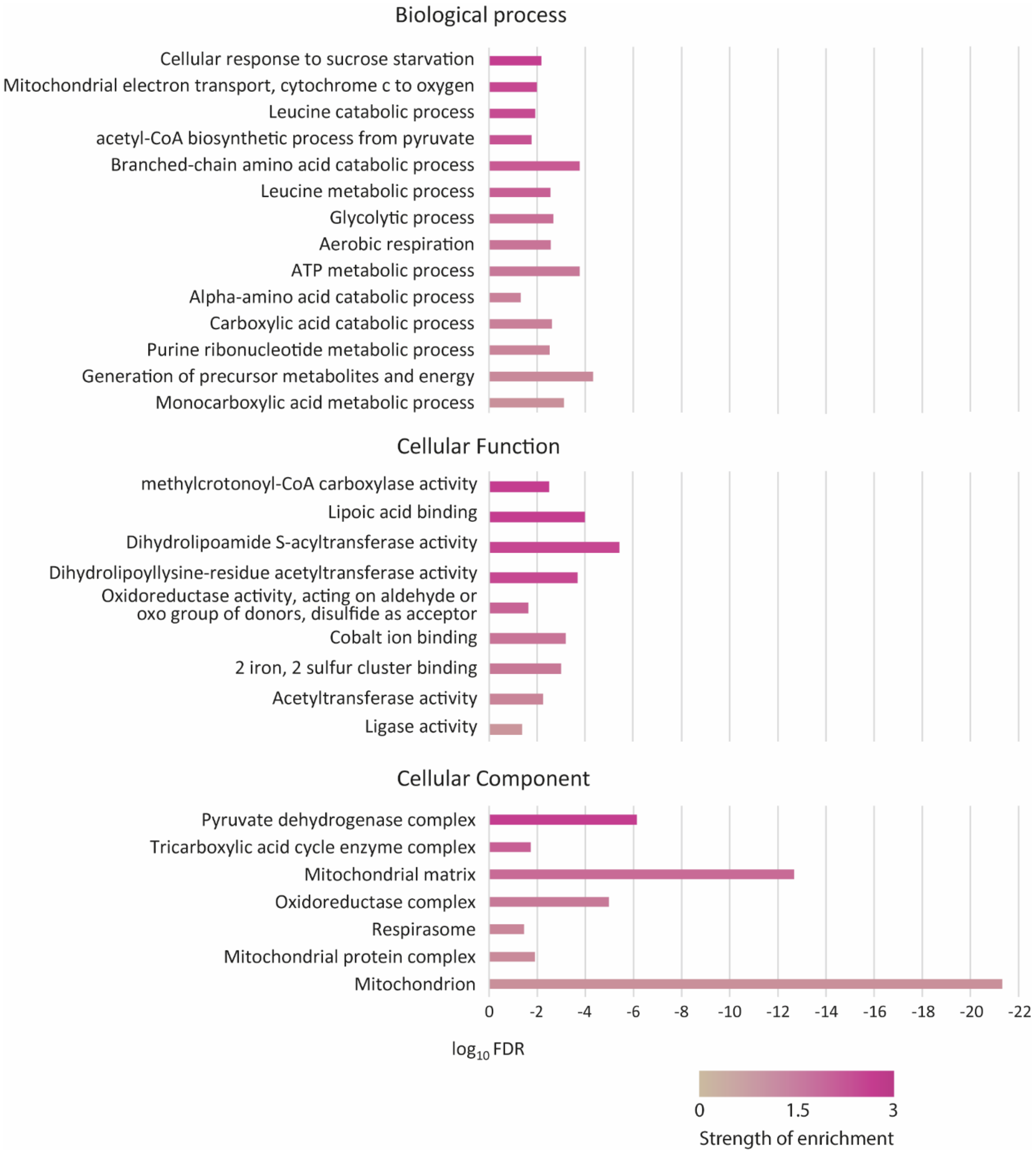

Figure S4: Functional enrichment analysis of guard cell-enriched proteins. 48 guard cell-enriched proteins (Δlog_2 FC <-1, identified in at least three replicates) were subjected to GO term enrichment analysis on String DB. Only GO terms with a strength of enrichment (log_{10}(observed/expected number of proteins in a random set)) >1 are shown.

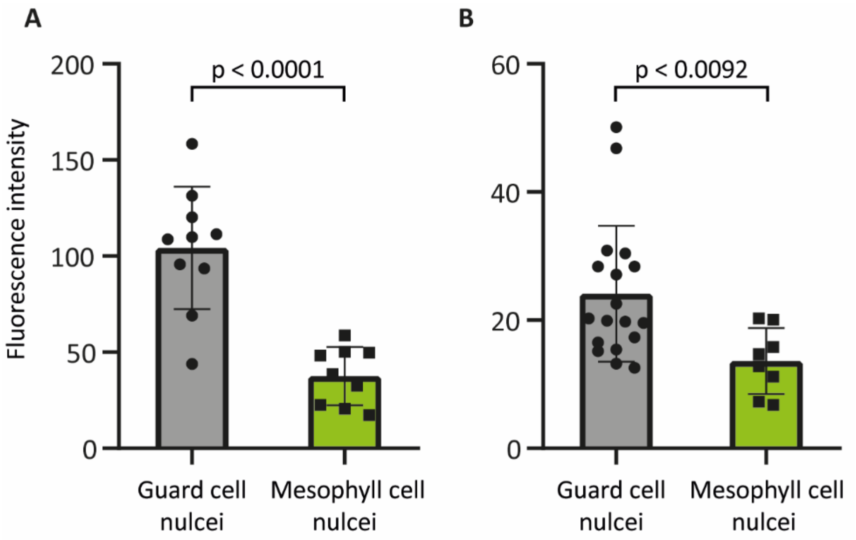

S5: Fluorescence intensity measurements of mesophyll versus guard cell derived signals in *pBCE2::NLS_tdTomato* and *pClpB-m::NLS_tdTomato* seedlings. 5-7-day-old Arabidopsis seedlings expressing the promoter-reporter construct for *pBCE2* (A) and *pClpB-m* (B) were analyzed. Pictures were converted to 8-bit and analyzed using ImageJ and mean grayscale. (A) Guard cell nuclei: N=10, Mesophyll cell nuclei: N=9, (B) **G**uard cell nuclei: N=18, Mesophyll cell nuclei: N=8.

Table S4: List of cell type specifically enriched mitoribosomal proteins identified in this data set. [1]states whether the candidate has paralogs and is part of a small gene family, [2]Δ log_2FC value from the mesophyll-guard cell comparison is given, with a value >1 showing mesophyll enrichment and <-1 guard cell enrichment.

Identifier	Name	[1]Small gene family	[2]Δ log_2FC mesophyll vs guard cell	References
At5g40080	ml41	Yes	4.17	Waltz *et al.* (2019), Tomal *et al.* (2019)
AT5g64650	bl17m	Yes	2.15	Waltz *et al.* (2019), Tomal *et al.* (2019)
AT4g23620	bL25mz	Yes	1.99	Tomal *et al.* (2019)
AT5g15980	ms81/RPPR8		1.61	Waltz *et al.* (2019), Waltz *et al.* (2020b)
AT1g77750	uS13m		1.44	Waltz *et al.* (2019)
AT4g30930	bL21m/NFD1		1.40	Waltz *et al.* (2019), Portereiko *et al.* (2006)
AT5g46160	uL14m/HLP	Yes	1.38	Waltz *et al.* (2019), Skinner *et al.* (2001)
AT5g02740			1.37	
AT1g61870	mS76/rPPR1		1.29	Waltz *et al.* (2019), Waltz *et al.* (2020b)
AT5g62270	mS75/GCD1		1.25	Waltz *et al.* (2019), Huang *et al.* (2017), Wu *et al.* (2012)
AT3g22300	uS10m/RPS10		1.19	Waltz *et al.* (2019), Kwasniak-Owczarek *et al.* (2019), Kwasniak *et al.* (2013), Majewski *et al.* (2009)
AT3g17465	uL3m		1.13	Waltz *et al.* (2019)
AT5g53070	bL9m		1.12	Waltz *et al.* (2019)
AT5g56940	bS16m		1.02	Waltz *et al.* (2019)
AT3g02650	mS80/rPPR6		-1.39	Waltz *et al.* (2019), Waltz *et al.* (2020b)
AT1g53645	mS84		-1.41	
AT4g28360	uL22m	Yes	-3.22	Waltz *et al.* (2019), Tomal *et al.* (2019)
